# Stimuli-Responsive Hydrogels of Poly(Methacrylic Acid)/Poly(N,N-dimethylacrylamide) Interpenetrating Polymer Networks as Drug Delivery Systems for Promethazine Hydrochloride

**DOI:** 10.3390/gels11040240

**Published:** 2025-03-25

**Authors:** Marin Simeonov, Ioanna Yildirim, Christo T. Tzachev, Elena Vassileva

**Affiliations:** 1 Laboratory on Structure and Properties of Polymers, Faculty of Chemistry and Pharmacy, University of Sofia, 1, J. Bourchier Blvd., 1164 Sofia, Bulgaria; evassileva@chem.uni-sofia.bg; 2 Faculty of Chemistry and Pharmacy, University of Sofia, 1, J. Bourchier Blvd., 1164 Sofia, Bulgaria; ioannayildirim@gmail.com (I.Y.); ohtct@chem.uni-sofia.bg (C.T.T.)

**Keywords:** hydrogels, interpenetrating polymer network, promethazine hydrochloride, oral delivery, smart materials, sustained drug delivery

## Abstract

Hydrogels with tunable properties are of great interest for the development of advanced drug delivery systems. In this study, novel hydrogels with an interpenetrating polymer network (IPN) structure were obtained from the pH-responsive poly(methacrylic acid) (PMAA) and the neutral poly(N,N-dimethylacrylamide) (PDMAM). The newly synthesized IPN hydrogels were shown to be pH responsive with a 1.5 to 2.5 fold increase in their equilibrium swelling ratio at a pH above 5 which makes them appropriate for targeted intestine drug delivery. Moreover, their pH responsiveness was found to be strongly influenced by the IPN’s composition. The IPN hydrogels were loaded with PMH via swelling and the drug entrapment efficiency was found to depend on their swelling characteristic varying with the IPN’s composition from 20% to 60%. The drug release profiles were investigated under conditions resembling the oral route of drug application. The PMH release profiles appeared to follow Fickian diffusion at a stomach-like pH = 1.2 and sub-diffusion mechanism at an intestine-like pH = 6.8. The results from this study reveal that IPN hydrogels of PMAA and PDMAM are promising candidates for oral delivery of promethazine hydrochloridee demonstrating pH responsiveness and controllable swelling dependent on their composition. Further investigations are planned to fully reveal their potential as smart drug delivery systems.

## 1. Introduction

Hydrogels are three-dimensional structures capable of absorbing and retaining large amounts of water without dissolving, which structural integrity is achieved through chemical and/or physical crosslinking [1]. Their unique structure and properties determine a remarkably wide spectrum of applications in the biomedical field [2]. Hydrogels can also be designed as smart materials capable of response to environmental changes such as pH, temperature, and electric or magnetic fields [3]. One of the most advanced types of polymeric networks, capable of forming hydrogels, are the interpenetrating polymer networks (IPN). They are composed of two or more polymer networks that interpenetrate into each other at molecular level, without forming covalent bonds between them [4] (Scheme S1, Supplementary Information). IPN hydrogels provide numerous advantages as drug delivery systems, including the ability to precisely control the drug release profile by adjusting the crosslinking density of both constituent networks, the formation of phase-separated structures by varying the hydrophilic/hydrophobic balance of the nature of their components, and programmable smart behavior (i.e., a predictable response to external stimuli changes, enabling on–off drug release). All these features allow for tailoring the drug release profile by simply varying the composition of the IPN [5]. 

Poly(methacrylic acid) (PMAA) is a pH-responsive polymer with the pKa of their pendant carboxylic group being around ~5.5. At a pH above its pKa, i.e. at neutral and alkaline conditions, these carboxylic groups (-COOH) are deprotonated and form carboxylate anions (-COO^−^) which results in enhanced swelling caused by the electrostatic repulsion between the similarly charged species [6]. This effect was shown to have a crucial role in the development of drug delivery systems for targeted intestinal drug delivery. At the low pH of the stomach (pH~1.2) which is below the PMMA pKa, the swelling of PMAA is limited, additionally hampered by the hydrogen bond formation between them, creating a denser network which slows down the drug release [3]. Under intestinal conditions where pH is around ~6.8, i.e. above the PMMA pKa, the transformation of the carboxylic groups into carboxylate anions causes enhanced swelling and thus a looser network, which favors the drug release into intestines [3]. This effect was demonstrated via the formation of PMMA IPN with hydrophilic polymers such as poly(acrylamide), where the ratio between the two IPN constituent networks determines the mechanism of the drug release [7]. Thus, PMAA is well suited as material for developing drug delivery vehicles targeted for the intestine.

Poly(N,N-dimethylacrylamide) (PDMAM) is a neutral, hydrophilic and biocompatible polymer [8]. Our previous studies demonstrate that when PDMAM is incorporated as a component into an IPN, it is able to control the overall swelling properties and drug release performance of the resulting IPN due to its hydrophilic nature [5]. Due to its hydrophilic nature, PDMAM could provide high swelling ratios of the drug delivery vehicles based on them, thus allowing for a rational control over the drug release profiles.

Promethazine hydrochloride (PMH) exhibits antihistaminic, sedative, and antiemetic activities. It is able to selectively block the peripheral H1 receptors, reducing in this way the histamine’s effects on the targeted cells. Moreover, it also inhibits the central histaminergic receptors, leading to depression of the reticular activating system and exhibits in this way sedative and hypnotic effects. Promethazine hydrochloride also demonstrates central anticholinergic activity and it is likely to mitigate nausea and vomiting by acting on the chemoreceptive trigger zone in the medulla [9]. When administrated orally, PMH is subjected to extensive first-pass metabolism which reduces its bioavailability to 25% [10]. Thus, other routes for its administration are sought to overcome this low bioavailability. The investigations on advanced PMH delivery systems include development of transdermal system [10], nasal gels [11,12], sublingual tablets [13], and complexation with β-cyclodextrin [14]. Another approach, demonstrated for other drugs with reduced bioavailability when administered orally, is their administration via using controlled release formulations [15]. A possible and unexplored until now approach to modify and prolong the PMH release profile in the gastro-intestinal tract (GIT) is the application of IPN as a drug delivery system. The advantages of IPN as drug delivery systems that could ensure prolonged and modified/smart drug release present an opportunity which is explored in the current study. For this purpose, we have chosen two components of the IPN, developed for the first time here, namely, (i) PMAA as a pH-sensitive polymer able to shrink at low pHs (as in the stomach) and to swell at neutral pHs (as in the intestines), preserving in this way the PMH release in the stomach and ensuring the targeted PMH release in the intestines; and (ii) PDMAM which is known to swell to a high extent ensuring in this way the full drug release at the targeted place, i.e. the drug dose needed for the proper cure could be delivered in the right place (intestines). Moreover, both polymers are known to be biocompatible, which is also another advantage important for the planned biomedical application.

The study is focused on the development of novel IPNs of PMAA and PDMAM and the exploitation of their potential for the modified and extended delivery of PMH in GIT. For this purpose, a series of IPNs are synthesized through the sequential method and PMAA/PDMAM IPN with different ratios between both constituents were obtained. These IPNs are characterized in terms of their swelling ability as well as their pH and temperature dependence. The synthesized IPNs are loaded with PMH and the respective drug entrapment efficiency is determined. The PMH release profiles from the IPNs are studied at two different pH values under conditions resembling oral application (stomach and intestines). The PMH release profiles are studied by using the main models for drug release kinetics.

## 2. Results and Discussion

### 2.1. Swelling Properties

#### 2.1.1. Equilibrium Swelling Ratio (ESR) in Water

**Figure 1:**
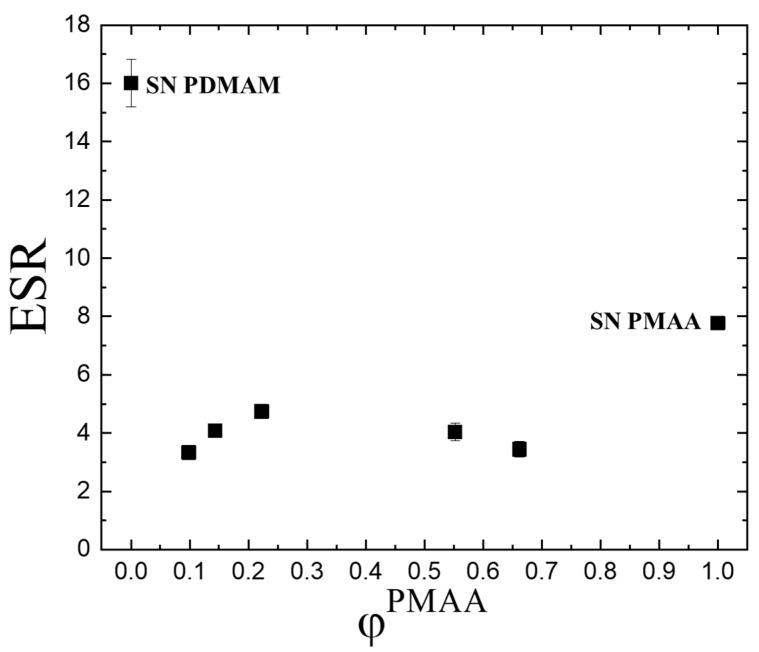
Equilibrium swelling ratio in water of SNs PMAA and PDMAM and their IPN as a function of PMAA weight fraction. Each datapoint is averaged from three different pieces from one sample (n = 3).

Figure 1 represents the ESR (calculated by applying equation 1, Materials and Methods) dependence of the PMAA/PDMAM IPN and single networks (SNs) of PMAA and PDMAM on the PMAA content in the respective network (φ^PMAA^). While SN PMAA has an ESR ~ 8, the SN PDMAM has double the ESR which is ~16. This difference could be related to several factors all contributing to the density of their networks:

(i) Different chemical crosslinking degrees as SN PDMAM is obtained by using 0.1 mol.% crosslinking agent, while SN PMAA is obtained via crosslinking with 4 mol.% of the same crosslinking agent. Thus, a denser network is expected for PMAA which limits the diffusion of the water molecules into it.
(ii) The pH of the media as PMAA is a pH-sensitive polymer. As distilled water was used as the swelling media, its pH is a little bit above and comparable to the pKa of the pendant carboxylic groups in PMAA which defines the possibility part of them to be in their neutral form while other part are ions. That means that the neutral carboxylic groups could form hydrogen bonds between themselves, thus forming an additional physical network on the top of the existing chemical one. At the same time, for PMMA, it is known that their side -CH_3_ groups which are directly bound to the macromolecular backbone could form via hydrophobic interactions between themselves small hydrophobic clusters which again act as physical network junctions. Thus, both factors work with a reduced swelling ability of PMAA as compared to PDMAM.

The ESR of the PMAA/PDMAM IPN is lower compared to the ESR for both SNs, being between 3 and 5, and does not show a clear dependence on the IPN’s composition, i.e. on the PMAA weight fraction (φ^PMAA^) (Figure 1). This reduces the ESR of the IPN to the respective SNs, which can be explained by (i) the increased IPN network density due to the mutual interlacing of PDMAM and PMAA networks, which penetrate into each other, thus increasing the overall density of the IPN (Scheme S2) and (ii) the formation of joint hydrophobic clusters between the side -CH_3_ groups from PMAA and the -N(CH_3_)_2_ groups from PDMAM, which make it denser and create more obstacles for the water diffusion media. We have observed similar effects of the IPN structure on the ESR in our previous work [3].

#### 2.1.2. pH-Responsiveness

**Figure 2:**
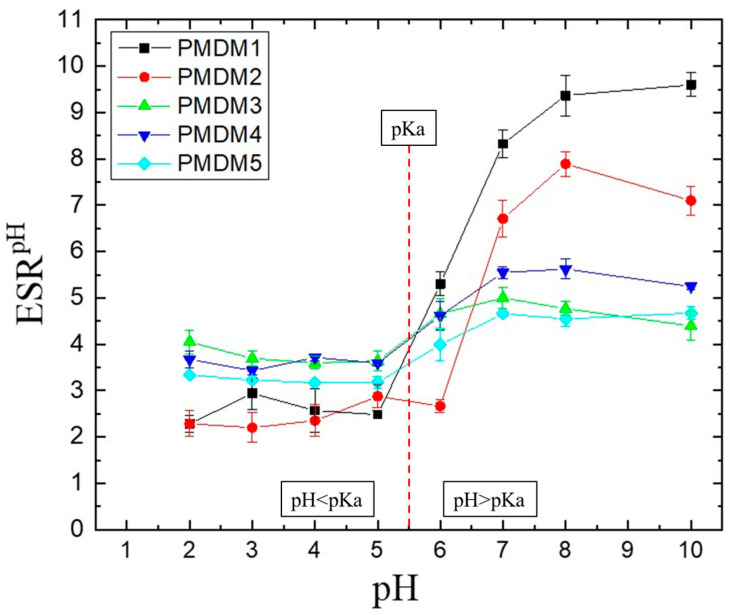
ESR dependence on pH for PMAA/PDMAM IPN: PMDM1 (φ^PMAA^ = 0.66), PMDM2 (φ^PMAA^ = 0.55), PMDM3 (φ^PMAA^ = 0.22), PMDM4 (φ^PMAA^ = 0.14), PMDM5 (φ^PMAA^ = 0.09). Each datapoint is averaged from three different pieces from one sample (n = 3). Each datapoint is averaged from three different pieces from one sample (n = 3).

The effect of pH on the ESR of the IPN is presented in Figure 2. As can be seen there, all IPNs show pH-dependent swelling expressed as a relatively sharp sigmoidal increase in their ESR (calculated by Equation (2), Section 4). We have determined the inflex point of the transition for all samples, obtained from the dependencies shown in Figure 2, and it appeared to be around a pH~6 (Table S1) which is very close to pKa of PMAA which is ~5.5 [16]. At a pH = 3, the IPN with higher PMAA content (i.e. with φ^PMAA^ = 0.66 and 0.55) swells less than the IPN with higher PDMAM content (φ^PMAA^ = 0.22, 0.14 and 0.09) exactly as it could be expected due to the hydrogen bond formation between COOH pendant groups of PMAA. At a pH = 7 and above, the ESR dependence on pH is “reverted” and the higher the φ^PMAA^ is, the higher the ESR value of the IPN is. This is due to the increase in the content of the ionizable component, namely PMAA. This effect is better illustrated in ta, where the ESRs of PMAA/PDMAM IPN at two different pHs, namely below (pH = 3, Figure S1A) and above (pH = 7, Figure S1B), and the pKa of PMAA are presented as a function of PMAA content.

This pH responsiveness is in line with the aimed release of the drug into intestines where pH is ~7 and thus the IPN will swell more and will release the drug while at the pH of the stomach will be shrunk and will not release the loaded drug. 

**Figure 3:**
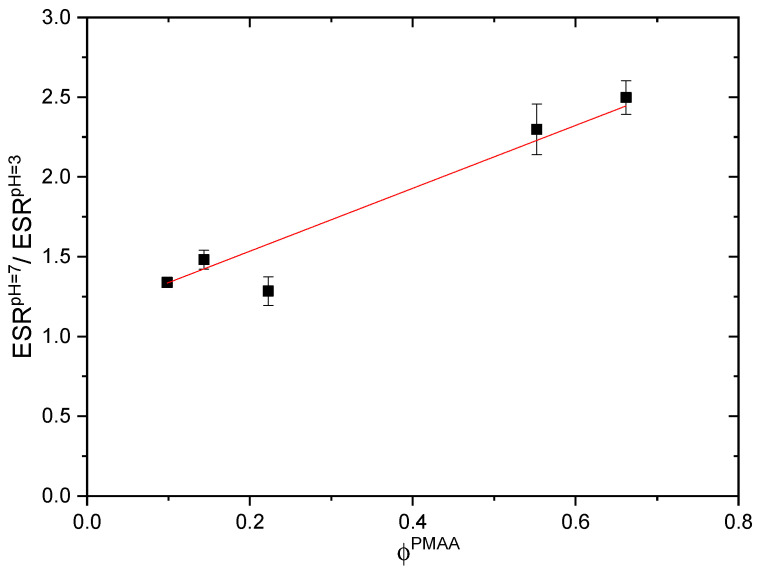
Ratio between ESR of PMAA/PDMAM IPN at two different pH values, namely pH = 3 and pH = 7. Red line represents the linear regression of the dependence. Each datapoint is averaged from three different pieces from one sample (n = 3).

We have also evaluated the ratio between the ESRs of all IPNs at these two pH values, namely at pH = 7 and pH = 3 (Figure S1, Supplementary Materials), and its dependence on the IPN’s composition is shown in Figure 3. This ratio increases linearly with increasing PMAA content (R^2^ = 0.9194) which means that the IPN composition could become a tool to control the drug release. The observed dependence is due again to the deprotonation of the pendant -COOH groups of PMAA turning them into carboxylate anions and enhancing the electrostatic repulsion between them and thus the drug release (Scheme S3, Supplementary Materials).

The electrostatic repulsion between the negative charges of PMAA increases the free volume within the polymer network, thus facilitating the diffusion of water molecules into it, resulting in an increased amount of water retained by the network. Such behavior of polymer carrier is known to be favorable for enteric drug delivery [17], similarly to commercially available pH-responsive methacrylate copolymers such as Eudragit^®^. Eudragit^®^ L100 Eudragit^®^ S100, differing at the methacrylic acid–methyl methacrylate ratio, are commonly utilized as pH-dependent coating agents to safeguard the drug core from the harsh gastric environment [18]. In our study, the specific design of PMAA/PDMAM IPN provides not just the possibility for designing a pH-responsive system, but also provides the possibility for control over the “intensity” of the pH-dependent swelling through altering the network density. This may contribute to the development of systems allowing drug delivery to a distal part of the colon for large molecules such as proteins and peptides [17].

#### 2.1.3. Temperature Responsiveness

**Figure 4:**
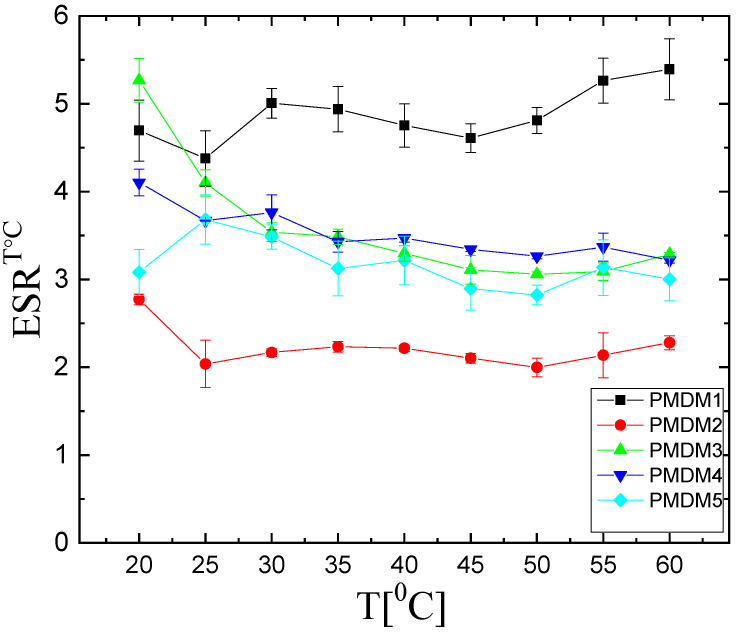
Temperature dependence of ESR of PMAA/PDMAM IPN in water. PMDM1 (φ^PMAA^ = 0.66), PMDM2 (φ^PMAA^ = 0.55), PMDM3 (φ^PMAA^ = 0.22), PMDM4 (φ^PMAA^ = 0.14), PMDM5 (φ^PMAA^ = 0.09). Each datapoint is averaged from three different pieces from one sample (n = 3). Each datapoint is averaged from three different pieces from one sample (n = 3).

The dependence of ESR (calculated by Equation (3), Section 4) of the PMAA/PDMAM IPN on temperature is presented in Figure 4. The IPN’ composition defines different abilities of the respective samples to respond to the temperature increase. The IPN with the highest PMAA content, namely PMDM1 (φ^PMAA^ = 0.66), shows the highest ESR for all temperatures but does not show clear temperature-responsive behavior as the ESR remains almost constant over the studied temperature range. This is the same for the behavior of both IPNs with the lowest PMAA content, namely PMDM4 (φ^PMAA^ = 0.14) and PMDM5 (φ^PMAA^ = 0.09), which also does not show a clear trend of decreasing its ESR as temperature increases (Table S1, F < Fcrit). The other two IPNs, namely PMDM2 (φ^PMAA^ = 0.55) and PDMAM3 (φ^PMAA^ = 0.22), show a slight decrease in their ESR as temperature increases, which is supported by ANOVA analysis (Table S1, F >> Fcrit). Thus, the studied IPNs with comparable content of both constituent SNs show a temperature dependence of their swelling behavior within the studied temperature region. This means that only some intermediate ratio between the IPN components could impart the ability of these materials to respond to the temperature variation. Their ESR temperature dependence resembles lower critical solution temperature (LCST) behavior where the main driving force is the hydrophobic/hydrophilic balance of the constituents. At low temperatures, the polymer is stabilized in the aqueous media (swollen in water hydrogel) mostly by strong hydrogen bonding between the polymer chains and the solvent (water in this case). As the temperature increases, these hydrogen intermolecular bonds are disrupted and the hydrophobic interactions between the polymer chains start to dominate over the hydrophilic polymer–solvent interactions, causing the polymer to collapse or aggregate [19]. In our study, PMAA has -CH_3_ side groups while PDMAM has -N(CH_3_)_2_ pendant groups which are hydrophobic and could give rise to hydrophobic interactions between both IPN constituent networks. As temperature increases, the hydrophobic character of these side groups starts to dominate and leads to the formation of hydrophobic clusters which act as a secondary physical network hindering the diffusion of water molecules and making the polymer network shrink. 

For polymers like PNIPAAM, where the hydrophobic nature of their pendant groups is more pronounced, LCST is seen as a sharp decrease in the polymer solubility in water at ~32 °C [5]. Here, such a sharp transition is not observed, but a gradual decrease in the PMDM2 (φ^PMAA^ = 0.55) and PDMAM3 (φ^PMAA^ = 0.22) ESR is observed. The less pronounced LCST could be explained by several factors such as the random distribution of the hydrophobic clusters [20] as well as by the IPN mutual interlacing which additionally limits the temperature effect [5]. The fact that this LCST-type behavior is observed only for certain ratios between the IPN’ components, is also in favor of the proposed joint formation of hydrophobic clusters between both IPN components.

### 2.2. Drug Loading

**Figure 5:**
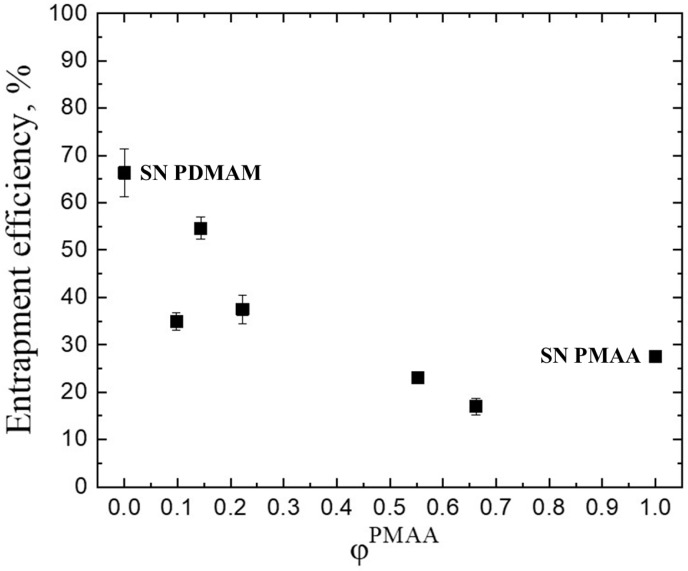
Entrapment efficiency with Promethazine hydrochloride of SNs PMAA and PDMAM and their IPN as function of PMAA weight fraction. Each datapoint is averaged from three different pieces from one sample (n = 3).

The negatively charged IPN under neutral conditions could be expected to interact easily with a cationic drug, so the electrostatic attraction could be a strong driving force to enhance the drug loading process. The results for the entrapment efficiency (EE) (calculated by Equation (4), Section 4) of the IPN presented in Figure 5 are, however, not in line with these considerations. The results show that another factor plays a stronger role here, namely the swelling ability of the polymer material. As the amount of the less swellable component, namely PMAA as seen from Figure 1, increases in the IPN, the EE of PMH in them decreases. This is in line with our previous research where the same dependence and significance of the swelling ability of the polymeric drug vehicle was observed for IPNs of PMAA and PAAM [16]. 

A reasonable explanation of this interplay between both factors expected to influence the drug loading process for the PMAA/PDMAM IPN is related to the conditions of the drug loading experiment: as the loading is performed in a solution of PMH with pH = 3.5–5.0 [21], which is below pKa of PMAA, its pendant -COOH groups are protonated, i.e. in their neutral form. This means that the amount of the drug which could be loaded in their hydrogels is determined mainly by the swelling capacity of these networks and the mechanism through which the drug is loaded is simple diffusion. This effect is similar to that observed in our previous works, where the drug loading in IPN composed only from non-ionizable polymers is governed by their swelling ability [3]. The component with higher swelling capacity—PDMAM (Figure 1) determines the higher entrapment efficiency at pH < pKa. Future studies with Fourier-transform infrared spectroscopy (FITR) and differential scanning calorimetry (DSC) are needed to clarify the molecular structure and to assess the possible polymer–drug interactions. As we demonstrated previously [3], the electrostatic attraction and hydrogen bonding between the cationic verapamil hydrochloride and the poly(methacrylic acid) proven by FTIR are the reason for the drug amorphization revealed by DSC [3]. Similar interactions are anticipated between the nitrogen atoms of PMH and the carboxylic groups of PMAA.

### 2.3. Drug Release

**Figure 6:**
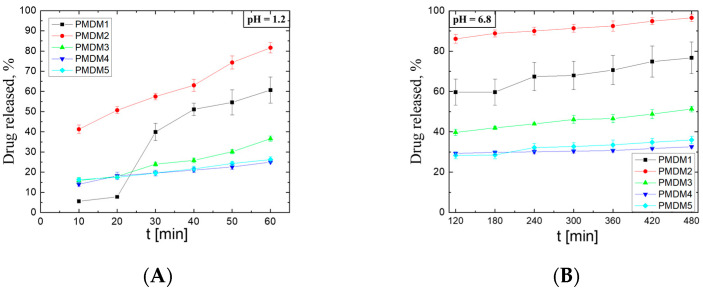
Drug release profiles of PMH from PMAA/PDMAM IPN at pH = 1.2 (**A**) and pH = 6.8 (**B**) for samples PMDM1 (φ^PMAA^ = 0.66), PMDM2 (φ^PMAA^ = 0.55), PMDM3 (φ^PMAA^ = 0.22), PMDM4 (φ^PMAA^ = 0.14), PMDM5 (φ^PMAA^ = 0.09). Each datapoint is averaged from three different pieces from one sample (n = 3).

We tested the drug release at two different pH values in order to mimic the GIT conditions. First, the IPNs loaded with PMH were immersed into acceptor media with a pH = 1.2 and after spending 2 h there, they were immediately transferred into accpetro media with a pH-6.8. The results from the drug release experiments performed at a pH = 1.2 and at pH = 6.8 are presented in Figure 6A,B. On the ordinates in both figures, the cumulative drug amount was released during the whole experiment (at both pHs, it is shown as a function of the total release time). The IPN PMDM2 (φ^PMAA^ = 0.55) demonstrates nearly a linear PMH release profile, which means that it ensures zero-order release kinetics under stomach-like conditions (Table 1). Despite the initial burst release of ~40%, PMDM2 shows the ability to linearly release a defined amount of PMH in which a linearity is also further observed for the intestine-like condition (Figure 6B) where the whole entrapped PMH is released to ~95% for 8 h.

On the contrary, the sample PMDM1 (φ^PMAA^ = 0.66) shows almost no burst effect but starts to release the PMH after the 20th min at a pH = 1.2 which release after the 30th min becomes almost linear. This linearity is also preserved when the sample is transferred to a pH = 6.8, releasing around 80% of PMH for 8 h. The jump in the drug release between 20th and 30th min could be explained with the PMH loading very close to (or underneath) the surface of the IPN sample instead of entering deep into the sample, ensuring an even distribution of the drug within the entire sample’s volume through diffusion. This explanation is in line with the explanation provided for the pH dependence of the entrapment efficiency and the reduced ability of the IPN to swell in PMH solution and is related to the formation of a physical network via hydrogen bonds formed between COOH pendant groups of PMAA.

The decreasing of the ionizable component (PMAA) content in PMDM3 (φ^PMAA^ = 0.22), PMDM4 (φ^PMAA^ = 0.14), and PMDM5 (φ^PMAA^ = 0.09) changes their drug release behavior significantly. They release the drug very slowly and gradually with no burst effect during the first 2 h in acidic media (Figure 6). The change in pH of the acceptor media does not change the linearity of the PMH release profile but the drug is released to no more than 45% for 8 h—an effect which could be partially due to the lowered solubility of PMH at a neutral pH. A similar effect was observed in an earlier study when chitosan hydrogels were used for PMH release [22], where the authors reported that PMH release from chitosan thin films was mainly controlled by the drug diffusion [23]. 

The kinetic models applied to the obtained experimental data show that PMH is released from the IPN hydrogels following zero-order release kinetics. This means that the drug release is independent of the drug concentration and is a function only of time, i.e., the IPN structure acts as a saturated reservoir ensuring stationary concentration gradient across a membrane [23]. As it can be seen from Table 1, the zero-order release constants (K_0_, Table 1) gradually decrease from 0.568 to 0.205 as φ^PMAA^ decreases. This can be attributed to the increased overall density of the IPN as higher amounts of the second components are introduced into the IPN, thus highlighting the importance of the specific IPN architecture. 

The results from the analysis with the Korsmeyer–Peppas model are also in good agreement for PMDM5 and PMDM3 as the values of the diffusional exponent n are 0.269 (PMDM5) and 0.461 (PMDM3), respectively. 

**Figure 7:**
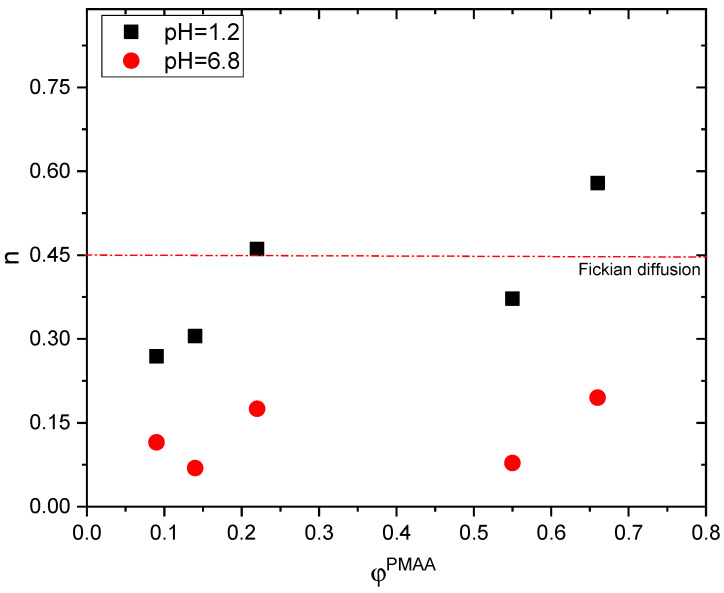
Diffusion exponent n of PMH released from PMAA/PDMAM IPN at pH = 1.2 (black squares) and pH = 6.8 (red circles) as evaluated by the Korsmeyer-Peppas model.

When the pH of the acceptor media is changed to pH = 6.8, the n values from the Korsmeyer–Peppas model dramatically decrease (Table 2, Figure 7) for all IPNs to n < 0.2, indicating a subdiffusion of PMH. We have obtained similar results in our previous work where PMAA/PAAM IPN hydrogels were applied as release systems for verapamil hydrochloride [3]. The drug sub-diffusion is a peculiar case of drug mass transport which is defined by (a) geometrical obstructions of the drug diffusion due to molecular crowding; (b) delays caused by the viscoelasticity of the media (hydrogel); and (c) incidental trapping of drug molecules into possible binding places [24]. All these factors are available in the PMAA/PDMAM IPN as the pendant –CH_3_ groups from both PMAA and PDMAM create hydrophobic clusters acting as physical barriers, i.e. geometrical obstructions, on the way-out of the drug from the hydrogels, thus retarding its release, similarly to the PMAA/PAAM IPN.

As it can be seen, the results from the test for in vitro drug dissolution are indicative for the potential of PMAA/PDMAM IPN as targeted and sustained drug delivery systems for cationic drugs such as promethazine hydrochloride. The optimal ratio between the two constituents provides a pH-responsive behavior and swelling capacity to maximize the drug dissolution in the gastric environment. One of the main drawbacks of PMH is its contraindications in cases of stomach ulcer, when applied orally [25]. Reducing the drug release in the stomach may be considered as a successful approach to overcome this problem and to allow for the administration of PMH in cases of such conditions. 

## 3. Conclusions

A series of novel PMAA/PDMAM IPN hydrogels was synthesized and evaluated as potential carriers for the oral delivery of PMH. These IPN hydrogels demonstrated a clear pH responsiveness and a non-sufficiently pronounced temperature responsiveness. When the pH of the media exceeds 5, the ionization of the pH-responsive PMAA leads to a 1.5- to 2.5-fold increase in the equilibrium swelling ratio of the hydrogels which demonstrates their suitability for intestinal targeted drug delivery. The entrapment efficiency of PMH was found to be defined by the swelling ability of the hydrogels and varies between 20% and 60%, depending on the IPN’s composition. PMH release profiles demonstrated a prolonged-release profile with up to 95% of the drug released within 8 hours depending on the IPN’s composition. The drug release kinetics models suggests a Fickian diffusion at pH = 1.2 and sub-diffusion of the drug at pH = 6.8. The findings of this study suggest that PMAA/PDMAM IPN hydrogels possess significant potential for oral drug delivery demonstrating strong pH responsive behavior. The full potential of these novel IPN hydrogels as advanced delivery systems for PMH will be further investigated by detailed biocompatibility assessment.

## 4. Materials and Methods

### 4.1. Materials

N,N;-dimethylacrylamide (DMAM, purum, 98.0%) was purchased from Fluka AG, Germany. Methacrylic acid (MAA, extra pure, 99.5%) was purchased by Across Organics, Belgium. 2-ketoglutaric acid(KGA) was purchased from Sigma-Aldrich and N,N-methylenebisacrylamide (MBAA) was purchased from Sigma-Aldrich. Promethazin hydrochloride (PMH) was provided by Knoll AG, Germany. All reagents were used as received without further purification.

### 4.2. IPN’s Synthesis

PMAA/PDMAM IPN hydrogels were synthesized following a two stage sequential method. 

Stage 1: single networks (SNs) of the poly(methacrylic acid) were obtained via free crosslinking radical UV polymerization. Briefly, the water solution of the monomer MAA (1.16 M), containing the crosslinking agent MBAA (4 mol.%, with respect to the monomer) and the initiator KGA (1.18 mol.%, with respect to the monomer), was irradiated by UV lamp (VILBER LOURMAT VL-4.L UV Lamp, 7 W) at 365 nm for 60 min (5 cm distance). Each of the synthesized SNs was thoroughly cleaned of all traces of the nonreacted compounds using distilled water (the wastewaters were checked daily by UV (Jasco V-730 UV/Vis spectrophotometer, Jasco, Japan).

Stage 2: the second (DMAM) network was in situ formed into the first SN to form PMAA/PDMAM IPN. This was accomplished by transferring dry PMAA SNs into DMAM aqueous solutions at varying concentrations. (from 0.5 M to 4 M) (Table 3), also containing MBAA (0.1 mol.% with respect to the monomer) and KGA (1.18 mol.% with respect to the monomer). After swelling for 72 h, the swollen SNs were placed for 60 min under a UV lamp at 365 nm for 60 min. In order to eliminate any remaining remnants of the non-reacted compounds, each of the produced IPNs was submerged in an excessive volume of distilled water (the wastewaters were checked daily by UV (Jasco V-730 UV/Vis spectrophotometer, Jasco, Japan). Following this procedure, IPNs with different compositions were established (weight fraction of PMAA) (Table 3). 

To ascertain the exact composition of the IPNs, two methods were used: (i) titrating the remaining methacrylic acid monomer in the waste waters obtained following the first stage, and (ii) determining the non-reacted DMAM in the final waste waters obtained following purification of the obtained IPN using the UV method for DMAM determination. More details for the synthetic procedure and the determination of the IPN’s composition are provided in the Supplementary Information (Table S2).

### 4.3. Swelling Properties

#### 4.3.1. Equilibrium Swelling Ratio 

The equilibrium swelling ratio (ESR) for all synthesized IPNs as well as SNs was determined in distilled water at temperature 24 ± 1 °C. Dry disk-shaped samples with a diameter of 4.5 mm were left to swell until they reached a constant weight. The ESRs were calculated by using the following equation:

(1) $$\mathrm{ESR}=\frac{m_{\mathrm{swollen}}-m_{\mathrm{dry}}}{m_{\mathrm{dry}}}$$

Here, $m_{\mathrm{swollen}}$ and $m_{\mathrm{dry}}$ are, respectively, the weights of the swollen sample andin its dry state. The data were averaged for at least three samples.

#### 4.3.2. pH Responsiveness

Similar to the method previously described, measurements were conducted at 24 ± 1 °C using various pH values between 2 and 10. In short, 4.5 mm diameter dry disk-shaped samples were swelled in buffer media with a predetermined pH. Each piece’s mass in its enlarged state was measured after it had reached equilibrium, usually after 24 h. Using the formula, the ESR was determined for every pH value:

(2) $$\mathrm{ESR}=\frac{m_{\mathrm{swollen}}^{\mathrm{pH}}-m_{\mathrm{dry}}}{m_{\mathrm{dry}}}$$

Here, $m_{\mathrm{swollen}}^{\mathrm{pH}}$ and $m_{\mathrm{dry}}$ are, respectively, the weights of the swollen piece at a certain pH and when dry. Buffer solutions, used throughout the experiment, were prepared following the procedure described elsewhere [26].

#### 4.3.3. Temperature Responsiveness

The equilibrium swelling ratio (ESR) was measured at various temperatures between 20 °C and 60 °C for every synthesized IPN. This was accomplished by allowing dry disk-shaped samples, measuring 4.5 mm in diameter, to swell in distilled water at a specific temperature. Their weight was obtained while they were swollen after they had stabilized (usually for five hours, as indicated by the fact that their weight had not changed). The ESR was computed using the following formula at a specified temperature:

(3) $$\mathrm{ESR}=\frac{m_{\mathrm{swollen}}^{T^{o}}-m_{\mathrm{dry}}}{m_{\mathrm{dry}}}$$

Here, $m_{\mathrm{swollen}}^{T^{o}}$ and $m_{\mathrm{dry}}$ are, respectively, the weights of the sample swollen at a certain temperature and in its dry state. The data were averaged for at least three samples.

### 4.4. Drug Loading 

Dry disk-shaped samples with a diameter of 8 mm were swollen for 72 h at 22 ± 1 °C in PMH water solution with a concentration of 100 mg/mL (pH of PMH solution = 4.5). After drying, the entrapment efficiency (EE) of PMH in each sample was calculated by using the following equation:

(4) $$\mathrm{EE}=\frac{m_{\mathrm{PMH}}^{0}-m_{\mathrm{PMH}}^{\mathrm{nonloaded}}}{m_{\mathrm{PMH}}^{0}}$$

Here, $m_{\mathrm{PMH}}^{0}$ and $m_{\mathrm{PMH}}^{\mathrm{nonloaded}}$ are respectively the initial amount of PMH in the solution and PMH left non loaded after swelling. 

The amount of free non-entrapped PMH was calculated by measuring the UV absorbance of the residual PMH solution after the loading process and appropriate dilution in 0.1 M HCl. For this purpose, a calibration curve of PMH in 0.1 M HCl (Equation (5)) was obtained by measuring the absorbance at 254 nm of a series of PMH aqueous solutions with concentrations of 0.002 ÷ 0.2 mg/ml. The linear regression of the PMH calibration curve in 0.1 M HCl is as follows:

(5) $${\mathrm{ABS}}_{1.2}=8.08\times C_{\mathrm{PMH}}+0.01\;\left(R^{2}=0.9999\right)$$

where $C_{\mathrm{PMH}}$ and ${\mathrm{ABS}}_{1.2}$ are, respectively, the PMH concentration, and its UV absorbance measured at 254 nm (for pH = 1.2).

### 4.5. In Vitro Drug Release 

The drug release was performed by using a dissolution test apparatus (PJ-3 Tablet Four-Usage Tester). The test was conducted in a 900 mL dissolution media that was kept at 37 ± 0.5 °C with a pH that changed during the release tests, while the paddle was rotated at a speed of 50 rpm. This was accomplished by immersing the PMH-loaded IPN in 0.1 mol L^–1^ HCl solution (pH 1.2) for 60 min, followed by a 24 h immersion in phosphate buffer solution (pH 6.8). Up to 24 h, 5 mL aliquots of the acceptor media were removed at predetermined intervals. The quantity of the drug in the sample solution was determined by UV spectroscopy at 254 nm (BOECO UV/Vis S-26 Spectrophotometer). The amount of the released PMH at pH = 1.2 was calculated using the calibration curve for PMH obtained in 0.1 M HCl (Equation (5)). The amount of the released PMH at pH = 6.8 was calculated using the calibration curve for PMH obtained in phosphate buffer (Equation (6)):

(6) $${\mathrm{ABS}}_{6.8}=12.31\times C_{\mathrm{PMH}}+0.01\;\left(R^{2}=0.9998\right)$$

where ${\mathrm{ABS}}_{6.8}$ is the UV absorbance measured at 254 nm (for pH = 6.8).

The average of three determinations was utilized in the data analysis, and the cumulative percentage of the drug release was calculated.

### 4.6. Drug Release Kinetics

The drug dissolution profiles was analyzed with the following equations:

- Zero order (ZO)
  (7) $$Q_{t}=Q_{0}+k_{0}\times t$$
- First order (FO)
  (8) $$logQ_{t}=logQ_{i}-k_{1}\times \frac{t}{2.303}$$
- Higuchi model (HM)
  (9) $$Q_{t}=k_{H}\times t^{0.5}$$
- Korsmeyer–Peppas model
  (10) $$\frac{Q_{t}}{Q_{\infty }}=k_{KP}\times t^{n}$$

Here, $Q_{0}$ is the initial amount of the drug presented in the dissolution media, $Q_{t}$ is the amount of drug dissolved at time *t*, *Q_i_* is the initial amount of drug in the samples, $Q_{\infty }$ is the total amount of drug expected to be released at infinite time, $k_{0}$, $k_{1}$, $k_{H}$, and $k_{KP}$ are kinetic constants, *t* is the time and *n* is the diffusional exponent that allows for determination the type of the drug release mechanism: when *n* ≤ 0.45, the drug release is realized through the Fickian diffusion mechanism; when 0.45 ≤ *n* ≤ 0.89, the drug release follows the abnormal (non-Fickian) diffusion mechanism; and when *n* > 0.89, the drug release follows a complex transport mechanism (super-case II transport). From the diffusion exponent values, taking into account the cylindrical geometry of the IPN samples, the diffusion coefficients of the PMH using the equation were obtained:

(11) $$D=\sqrt[{n}]{\frac{k_{KP}}{4}}\pi l^{2}$$

where $k_{KP}$ is the same from Equation (10), *p* is the Ludolphine number (*π* = 3.14), *l* is the sample height (in m), and *n* is the diffusion exponent from Equation (10).

## Figures and Tables

**Table 1:** Kinetics models applied to the PMH release profiles obtained within this study for PMAA/PDMAM IPN at pH = 1.2.

pH = 1.2		PMDM1	PMDM2	PMDM3	PMDM4	PMDM5
**Zero order**	**K_0_**	0.568	0.552	0.454	0.328	0.205
	**R_0_^2^**	0.932	0.992	0.975	0.962	0.991
**First order**	**K_1_**	3.3 × 10^−1^	1 × 10^−2^	2.4 × 10^−3^	1.1 × 10^−3^	1.1 × 10^−3^
	**R_1_^2^**	0.932	0.956	0.965	0.964	0.989
**Higuchi**	**K_H_**	4.378	6.010	4.902	3.639	2.216
	**R_H_^2^**	0.950	0.973	0.939	0.979	0.956
**Korsmeyer–Peppas**	**K_KP_**	0.0567	0.1679	0.0501	0.0698	0.0824
	**n**	0.579	0.372	0.461	0.305	0.269
	**R_KP_** **^2^**	0.950	0.969	0.935	0.984	0.920
	**D^1.2^ (m^2^/s)**	2.02 × 10^−06^	6.24 × 10^−10^	2.35 × 10^−10^	5.4 × 10^−12^	1.69 × 10^−12^

**Table 2:** Kinetics models applied to the PMH release profiles obtained within this study for PMAA/PDMAM IPN at pH = 6.8.

pH = 6.8		PMDM1	PMDM2	PMDM3	PMDM4	PMDM5
**Zero order**	**K_0_**	0.0259	0.0184	0.0340	0.0117	0.0227
	**R_0_^2^**	0.934	0.977	0.985	0.942	0.919
**First order**	**K_1_**	8.1 × 10^−4^	1.6 × 10^−3^	2 × 10^−4^	4 × 10^−5^	1 × 10^−4^
	**R_1_^2^**	0.937	0.968	0.983	0.944	0.923
**Higuchi**	**K_H_**	0.828	0.618	1.125	0.453	0.725
	**R_H_^2^**	0.948	0.978	0.981	0.887	0.950
**Korsmeyer–Peppas**	**K_KP_**	0.225	0.591	0.169	0.208	0.183
	**n**	0.195	0.078	0.175	0.069	0.115
	**R_KP_** **^2^**	0.926	0.961	0.971	0.839	0.937
	**D^6.8^ (m^2^/s)**	1.22 × 10^−12^	7.08 × 10^−17^	4.41 × 10^−14^	7.73 × 10^−25^	7.05 × 10^−18^

**Table 3:** SNs of PMAA and PDMAM and their IPN expressed as weight fraction of PMAA (φ^PMAA^).

Sample	SN PMAA	PMDM1	PMDM2	PMDM3	PMDM4	PMDM5	SN PDMAM
**C_DMAM_ [mol L^−1^]**	0	0.5	1	2	3	4	1
**φ^PMAA^**	1	0.66	0.55	0.22	0.14	0.09	0

## Data Availability

The raw/processed data required to reproduce these findings cannot be shared at this time as the data also form part of an ongoing study.

## Supplementary Materials

The following supporting information can be downloaded at https://www.mdpi.com/article/10.3390/gels11040240/s1, Scheme S1. The phase-separated structure of IPNs, obtained via sequential method. The first network (in blue) is obtained and then in situ the second network (in red) is formed. The IPN consists of two mutually interlaced networks, where the second network forms domains within the first network, i.e., the IPN has a phase-separated structure. Scheme S2. The increase in the overall network density of PMAA/PDMAM IPN as the amount of the second network of PDMAM increases (respectively r^PMAA^ decreases). Scheme S3. Hydrogen bond formation (blue dotted lines) at pH < pKa of the COOH groups of PMAA and electrostatic repulsion (red arrows) due to their ionization at pH > pKa.; Figure S1. ESR as a function of φPMAA of PMAA/PDMAM IPNs at pH = 3(A) and pH = 7(B).; Table S1. Analysis of Variance (ANOVA) of the data obtained for temperature responsiveness of PMAA/PDMAM IPN.; Table S2. Conversion of DMAM to PDMAM.

## Abbreviations

The following abbreviations are used in this manuscript:

IPN	Interpenetrating polymer network(s)
SN	Single network(s)
PMAA	Poly(methacrylic acid)
PDMAM	Poly(N,N-dimethylacrylamide)
PMH	Promethazine hydrochloride
ESR	Equilibrium swelling ratio
EE	Entrapment efficiency

## References

[1] Liu B., Chen K. (2024). Advances in Hydrogel-Based Drug Delivery Systems. Gels.

[2] Stanciu L., Diaz-Amaya S. (2022). Composite Biomaterials.

[3] Simeonov M., Kostova B., Vassileva E. (2016). Interpenetrating Polymer Networks of Poly (Methacrylic Acid) and Polyacrylamide: Synthesis, Characterization and Potential Application for Sustained Drug Delivery. RSC Adv..

[4] Jenkins A.D., Kratochvíl P., Stepto R.F.T., Suter U.W. (1996). Glossary of Basic Terms in Polymer Science (IUPAC Recommendations 1996). Pure Appl. Chem..

[5] Simeonov M., Kostova B., Mihaylova R., Vassileva E. (2025). Hydrogels of Poly (2-hydroxyethyl methacrylate) and Poly (N,N-dimethylacrylamide) Interpenetrating Polymer Networks as Dermal Delivery Systems for Dexamethasone. Pharmaceutics.

[6] Bhattacharjee S., Goswami S., Das S., Bhattacharjee S., Bhaladhare S. (2023). pH-Responsive, Stable, and Biocompatible Functional Nanogels Based on Chitosan (CS)/Poly Methacrylic Acid (PMAA) Polymers: Synthesis and Characterization. Mater. Today Commun..

[7] Simeonov M., Monova A., Kostova B., Vassileva E. (2017). Drug Transport in Stimuli Responsive Acrylic and Methacrylic Interpenetrating Polymer Networks. J. Appl. Polym. Sci..

[8] Mann J.L., Grosskopf A.K., Smith A.A.A., Appel E.A. (2021). Highly Branched Polydimethylacrylamide Copolymers as Functional Biomaterials. Biomacromolecules.

[9] National Cancer Institute Promethazine Hydrochloride. https://www.cancer.gov/publications/dictionaries/cancer-drug/def/promethazine-hydrochloride.

[10] Shirisha S., Sahoo S.K., Veena T., Rao Y.M. (2024). Design and Evaluation of Different Transdermal Therapeutic Systems of Promethazine Hydrochloride. Ind. J. Pharm. Educ..

[11] Boraste S.V., Patil S.B. (2024). Formulation Development and Evaluation of Nasal in Situ Gel of Promethazine Hydrochloride. Drug Dev. Ind. Pharm..

[12] McDonough J.A., Persyn J.T., Nino J.A., Dixon H., Boland E.J., Wang Z., Putcha L. (2007). Microcapsule-Gel Formulation of Promethazine HCl for Controlled Nasal Delivery: A Motion Sickness Medication. J. Microencapsul..

[13] Alyami H.S., Ibrahim M.A., Alyami M.H., Dahmash E.Z., Almeanazel O.T., Algahtani T.S., Alanazi F., Alshora D.H. (2021). Formulation of Sublingual Promethazine Hydrochloride Tablets for Rapid Relief of Motion Sickness. Saudi Pharm. J..

[14] Shah J.N., Shah K.N., Mehta T.A. (2015). Hydroxy Propyl β-Cyclodextrin Complexation of Promethazine Hydrochloride for the Formulation of Fast Dissolving Sublingual Film: In Vitro and in Vivo Evaluation. J. Pharm. Investig..

[15] Vismari L., Pires M.L.N., Benedito-Silva A.A., Calil H.M. (2002). Bioavailability of Immediate and Controlled Release Formulations of Lithium Carbonate. Rev. Bras. Psiquiatr..

[16] Zhang J., Peppas N.A. (2001). Molecular Interactions in Poly (Methacrylic Acid)/Poly (N-isopropyl Acrylamide) Interpenetrating Polymer Networks. J. Appl. Polym. Sci..

[17] Maroni A., Moutaharrik S., Zema L., Gazzaniga A. (2017). Enteric Coatings for Colonic Drug Delivery: State of the Art. Expert Opin. Drug Deliv..

[18] Gvozdeva Y., Staynova R. (2025). pH-Dependent Drug Delivery Systems for Ulcerative Colitis Treatment. Pharmaceutics.

[19] Heskins M., Guillet J.E. (1968). Solution Properties of Poly (N-Isopropylacrylamide). J. Macromol. Sci. Chem..

[20] Mueller K.F. (1992). Thermotropic Aqueous Gels and Solutions of N,N-Dimethylacrylamide-Acrylate Copolymers. Polymer.

[21] National Center for Biotechnology Information PubChem Compound Summary for CID 6014, Promethazine Hydrochloride. https://pubchem.ncbi.nlm.nih.gov/compound/Promethazine-hydrochloride.

[22] Seki Y., Yurdakoc K. (2008). Synthesis of pH Dependent Chitosan-EPI Hydrogel Films and Their Application Forin Vitro Release of Promethazine Hydrochloride. J. Appl. Polym. Sci..

[23] Bruschi M.L. (2015). Mathematical Models of Drug Release. Strategies to Modify the Drug Release from Pharmaceutical Systems.

[24] Miyaguchi T., Akimoto T. (2011). Intrinsic Randomness of Transport Coefficient in Subdiffusion with Static Disorder. Phys. Rev. E Stat. Nonlin. Soft Matter Phys..

[25] Mayo Clinic Promethazine (Oral Route). https://www.mayoclinic.org/drugs-supplements/promethazine-oral-route/description/drg-20070609.

[26] Pourjavadi A., Mahdavinia G.R. (2006). Superabsorbency, pH-Sensitivity and Swelling Kinetics of Partially Hydrolyzed Chitosan-g poly (Acrylamide) Hydrogels. Turk. J. Chem..

