# Supplementary material for: Stimuli-Responsive Hydrogels of Poly(Methacrylic Acid)/Poly(N,N-dimethylacrylamide) Interpenetrating Polymer Networks as Drug Delivery Systems for Promethazine Hydrochloride
Source: Gels. 2025 Mar 25;11(4):240. doi: 10.3390/gels11040240 (PMC12027187; doi:10.3390/gels11040240)
Supplement: Supplementary file 1 [file gels-11-00240-s001.zip › gels-3480964-supplementary.pdf]

Supplementary information

Stimuli responsive hydrogels of poly(methacrylic acid)/poly(dimethylacrylamide) interpenetrating polymer networks as drug delivery systems for promethazine hydrochloride

Marin Simeonov[1,*], Ioanna Yildirim [2], Christo Tzachev[2] and Elena Vassileva[1]

[1] Laboratory on Structure and Properties of Polymers, Faculty of Chemistry and Pharmacy, University of Sofia, 1, J. Bourchier Blvd., 1164 Sofia, Bulgaria; m.simeonov@chem.uni-sofia.bg (M.S); evassileva@chem.uni-sofia.bg (E.V).
[2] Faculty of Chemistry and Pharmacy, University of Sofia, 1, J. Bourchier Blvd., 1164 Sofia, Bulgaria; ioannayildirim@gmail.com *(Y.Y.);* ohtct@chem.uni-sofia.bg (C.T)
* Correspondence to: m.simeonov@chem.uni-sofia.bg

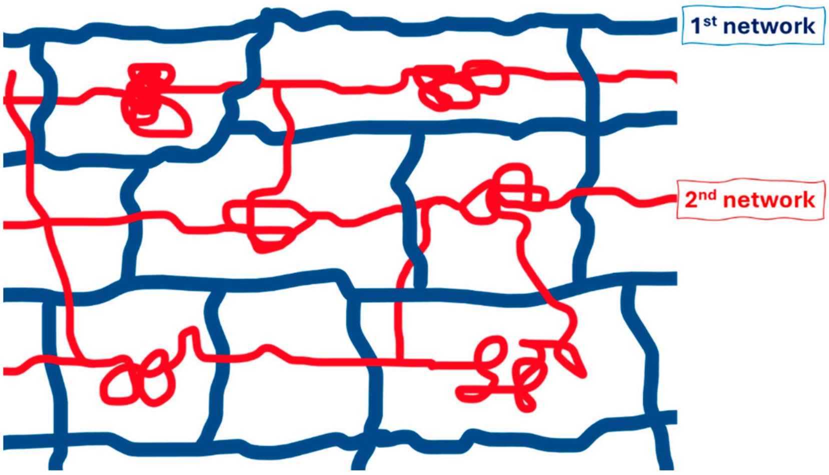

Scheme S1. The phase separated structure of IPNs, obtained via sequential method. The 1st network (in blue) is obtained and then in situ the 2nd network (in red) is formed. The IPN consists of two mutually interlaced networks, where the 2nd network forms domains within the 1st network, i.e. the IPN has a phase separated structure.

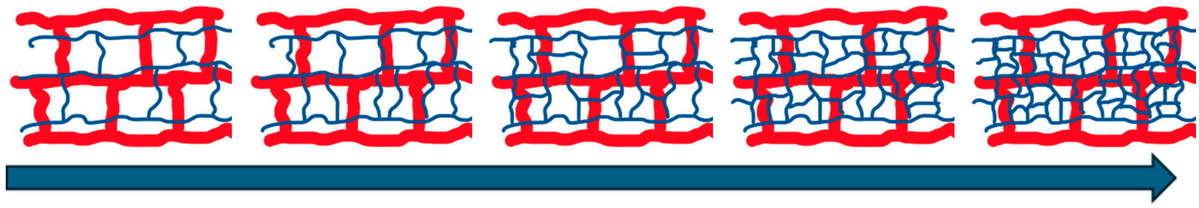

Scheme S2. The increase in the overall network density of PMAA/PDMAM IPN as the amount of the 2nd network of PDMAM increases (respectively r^{PMAA} decreases).

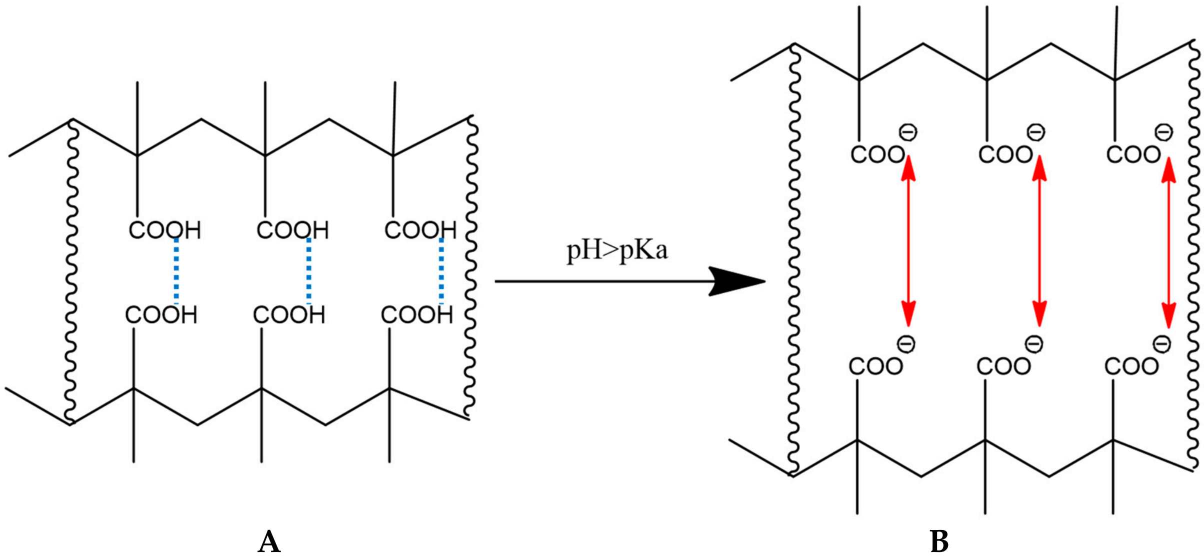

Scheme S3. Hydrogen bonds formation (blue dotted lines) at pH<pKa of the COOH groups of PMAA (A) and electrostatic repulsion (red arrows) due to their ionization at pH>pKa (B).

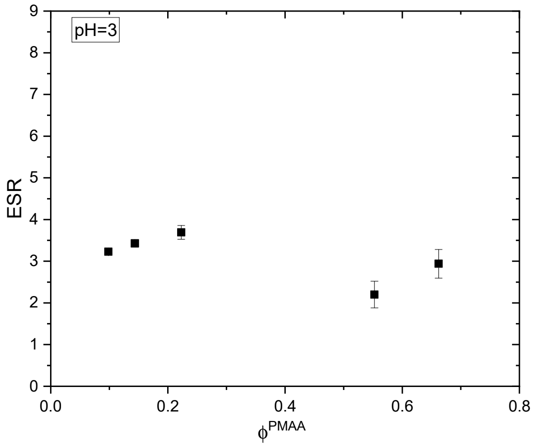

A

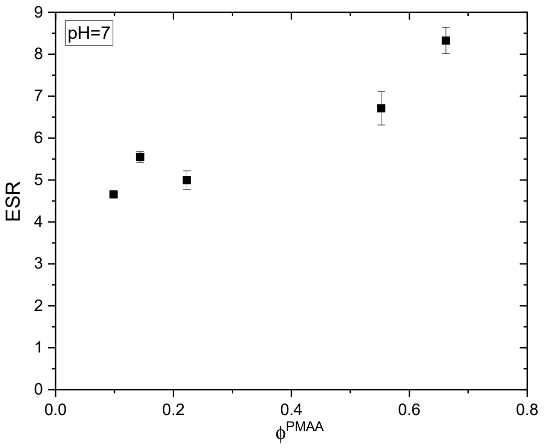

B

Figure S1. ESR as a function of φ^{PMAA} of PMAA/PDMAM IPNs at pH=3(**A**) and pH=7(**B**).

Table S1. Analysis of Variance (ANOVA) of the data obtained for temperature responsiveness of PMAA/PDMAM IPN.

**Sample**	**F**	**p-value**	**F crit**
**PMDM1**	0.21	0.98	2.51
**PMDM2**	0.11	0.99	2.51
**PMDM3**	31.40	4.88×10^{-9}	2.51
**PMDM4**	6.78	3.79×10^{-4}	2.51
**PMDM5**	1.38	0.27	2.51

Data is analysed by ANOVA at level of statistical significance p=0.05.

Determination of the IPN's composition

The conversion of MAA to PMAA was evaluated by applying a standard titration procedure. To this purpose, 5 ml aliquot from the collected wastewaters, used for purifying of one SN PMAA, was titrated by a standard aqueous solution of NaOH (0.105 N, determined by titration with HCl) and the quantity of the non-reacted MAA was calculated. The conversion of MAA to PMAA was determined to be 88% ±2% for all synthesized SNs PMAA.

The non-reacted DMAM quantity was determined in order to obtain the exact IPN's composition. To this purpose three independent series of DMAM water solutions in the concentration range 1÷6 ppm were prepared and their UV absorbance was measured at 234 nm. From the obtained date a calibation curve of DMAM was obtained:

$$\mathbf{ABS_{DMAM} = 0.0046 \times C_{DMAM} + 0.0534\ (R^2 = 0.9980)}\ \text{(S1)}$$

The conversion of DMAM to PDMAM was determined to be as shown in Table S1.

Table S2. Conversion of DMAM to PDMAM

Sample	C_{DMAM}	Conversion [%]
**PMDM1**	0.5	85
**PMDM2**	1	88
**PMDM3**	2	92
**PMDM4**	3	95
**PMDM5**	4	98
